# Management of Parapharyngeal Space Tumors: Clinical Experience with a Large Sample and Review of the Literature

**DOI:** 10.3390/curroncol30010078

**Published:** 2023-01-11

**Authors:** Chuanya Jiang, Wenqian Wang, Shanwen Chen, Yehai Liu

**Affiliations:** 1 Department of Otorhinolaryngology—Head and Neck Surgery, The First Affiliated Hospital of Anhui Medical University, Hefei 230022, China; 2 Department of Otorhinolaryngology—Head and Neck Surgery, Wuhu Hospital, East China Normal University, Wuhu 241001, China

**Keywords:** parapharyngeal space tumors, surgical management, endoscopy-assisted technique

## Abstract

Parapharyngeal space (PPS) tumors are rare, and they account for 0.5–1.5% of all head and neck tumors. This study summarized the findings of large-sample clinical studies of PPS tumors and reported the clinical work-up and management of 177 cases of PPS tumors at our center. This retrospective study included patients treated for PPS tumors between 2005 and 2020 at our center. The basic characteristics, symptoms, surgical approach, complications, and recurrence rates were analyzed. A total of 99 male and 78 female patients, with a mean age of 48.3 ± 15.1 years, were enrolled in this study. The most common symptoms were external or intraoral masses (114 patients, 64%). Surgical management leveraging, a cervical approach, was used for 131 cases (74%). The tumors were benign for 92% (160 cases), with pleomorphic adenoma being the most common (88 cases, 50%). Surgical complications were reported for 31 cases (18%); facial and vocal cord paralyses were the most common. Three cases of recurrence were observed during the follow-up. PPS tumors are rare and present with atypical clinical manifestations. The current study, which involved cases in a large single center, demonstrates the importance of surgical interventions for PPS tumors. The use of endoscopic techniques has further expanded the scope of traditional surgical approaches and demonstrated its advantages in selected cases.

## 1. Introduction

The parapharyngeal space (PPS) is a deep and complex area of the lateral neck. It is located on the pharyngeal side, showing an inverted triangle up to the skull base and down to the hyoid bone level. The fascia, extending backward from the styloid process to the tensor veli palatine muscle, divides the PPS into the anterior and posterior compartments. The anterior (styloid process anterior) space contains fat and salivary tissue and is located behind the pterygoid muscle. The posterior (styloid process posterior) space contains the carotid artery and internal jugular vein, cranial nerves IX, X, and XII, and the lymph gland [1,2]. This is conducive to a reasonable prediction of the pathology of the primary tumor in this region. At the same time, it leads to significant differences in the surgical approach and difficulty of the operation.

Primary tumors in the PPS account for 0.5–1% of head and neck tumors, most of which are benign (80%) [3,4]. Radiotherapy and chemotherapy are available; however, surgical resection is still the main treatment for PPS tumors based on the consideration of the general condition of patients and tumor types. Due to the development of endoscopic technology and imaging, surgical approaches for PPS lesions have been extended. The increasing demand for beauty has promoted minimally invasive and aesthetic surgical procedures for this area [5,6]. The classical surgical approaches are mainly external, and they include transcervical, transparotid, transcervical-parotid, and mandibular split approaches. Emerging approaches include natural cavity approaches, such as endoscopic-assisted transoral or transnasal approaches. This surgical method has high requirements of mastery of endoscopic technology and the anatomy of the primary tumor site; therefore, it is mostly used for highly selective cases [7].

Riffat et al. [3] and Kuet et al. [4] conducted a systematic review of PPS tumor management in 2014 and found that the number of cases in most studies was small. On the one hand, the incidence of PPS tumors is low, and on the other hand, the complexity of surgery leads to the concentration of patients with PPS tumors in tertiary centers. The immaturity of surgical technology may lead to an increase in the rates of complications and recurrence; therefore, studies with large samples should better reflect the true treatment outcomes.

There is new research on this topic since two reviews were published. However, the sample size of these studies was small, and similar studies with large samples are lacking [8,9,10,11,12,13,14,15]. There is a large number of patients in China; therefore, Chinese studies on related topics have also been widely reported. The purpose of this study was to report our experience with the treatment of PPS lesions at our center and summarize the findings of previous large-sample studies on PPS tumor management.

## 2. Materials and Methods

The medical records of consecutive patients diagnosed with PPS tumors at our center between 2005 and 2020 were retrospectively examined. Follow-up data were obtained using the contact information of all patients through clinical record notes. Data on the general characteristics of the patients and lesions, surgical methods, and prognosis were collected. This study was approved by the Institutional Review Board (IRB). The requirement for written informed consent was waived because the patient data were de-identified.

**Figure 1:**
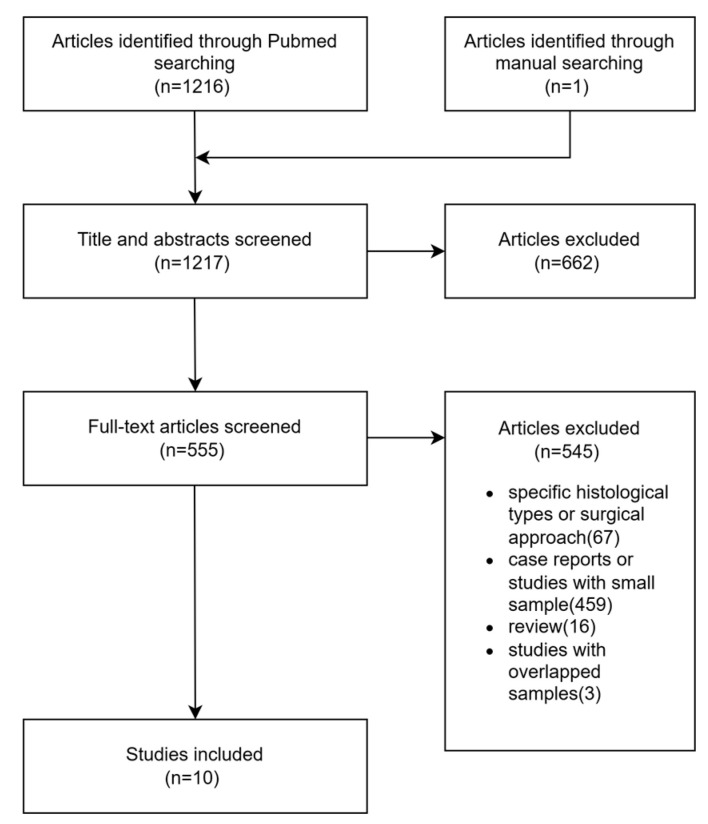
Flow diagram of the selected studies.

We also conducted a review in September 2022. MEDLINE (PubMed) database was searched for articles published between 1988 and 2022 using the search term “parapharyngeal.” We did not search the Chinese database because high-quality Chinese journals related to PPS were also included in PubMed. There have been some systematic reviews on PPS tumors. Therefore, only reports of studies with larger samples (>100 cases) evaluating tumors primarily originating from the PPS were included in the current study. Studies involving non-human subjects and those focusing on tumor pathology or diagnostic techniques were excluded. For studies by the same team, only one was selected. The flow diagram of selected studies is shown in Figure 1.

## 3. Results

**Figure 2:**
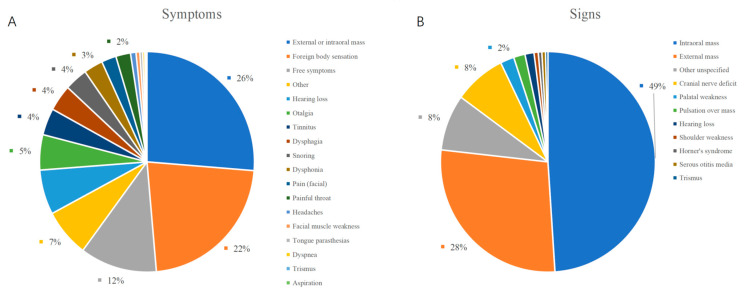
Distribution of the symptoms and signs of PPS lesions. (**A**) symptoms; (**B**) signs.

A total of 99 male and 78 female patients aged between 5 and 81 years (mean ± SD, 48.3 ± 15.1 years) were enrolled in this study. The most common symptoms for PPS lesions were external or intraoral masses (114 cases, 64%) and pharyngeal foreign body sensation (15 cases, 8%); 19 cases (11%) were asymptomatic. Other uncommon symptoms and results of previous studies with large sample sizes are listed in Table 1 [16,17,18,19,20]. In our group, the majority of patients had a self-reported neck or oropharyngeal mass; some patients did not have any clinical manifestations. Swelling of the neck and oropharynx was the most common finding on physical examination. Other rare findings such as trismus and otitis media were rarely mentioned [16,18]. The results are summarized in Table 1 and Figure 2.

**Figure 3:**
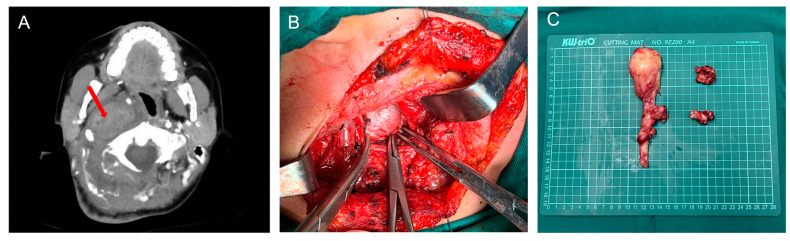
(**A**) Axial view of enhanced CT of a neurofibroma (red arrow); (**B**) Perioperative image of lesion removal through a transcervical approach; (**C**) Specimen origin from the vagus nerve.

**Figure 4:**
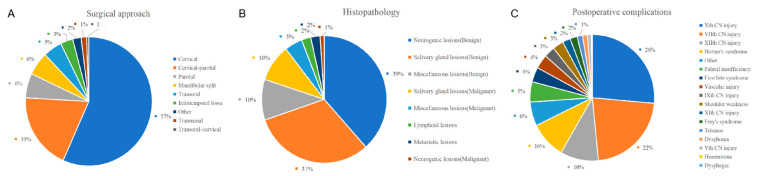
(**A**) Distribution of the different surgical approach; (**B**) Distribution of histopathology; (**C**) Distribution of complications.

Computed tomography, magnetic resonance, and angiography were used to diagnose PPS lesions. At our center, computed tomography is routinely performed preoperatively for all patients, and magnetic resonance is scheduled if needed. Cytological pathology biopsies were performed less frequently. Other examinations, such as laryngoscopy, ultrasound, and hearing examinations, were arranged according to the needs of the patient. Of the 177 patients, 175 underwent surgery; an example is shown in Figure 3. Table 2 [16,17,18,19,20,21,22,23,24] and Figure 4A summarize the investigations for surgical approach. Two patients who did not undergo surgery were transferred to another hospital or were administered chemoradiotherapy. Of the patients who received surgical treatment, the transcervical approach was the most preferred surgical method for 131 (75%), and the transcervical-parotid and transoral approaches were used for 11 (6%) and 20 (11%) cases, respectively. Other approaches included the transnasal, mandibular split, and combined approach. The transnasal approach was used for one patient who was previously treated with PPS tumor resection via the transcervical approach. The frequency of use of the transoral or transnasal surgery is not high because of the higher technical requirements for the scope of the lesion and surgeon [25,26].

A summary of the histopathological results is shown in Table 3 [16,17,18,19,20,21,22,23,24,27] and Figure 4B. The pathological types of PPS lesions were diverse. Benign tumors accounted for the majority of the cases in our group, with only 15 (8%) patients having malignant tumors. The most common benign lesions were pleomorphic adenomas and schwannomas. Malignant tumors have various pathological types. The overall postoperative complication rate was 18% (N = 31). The most common complications were hoarseness and facial nerve palsy. Other postoperative complications included tongue extension deviation, Horner’s syndrome, and trismus. The planned nerve sacrifice was not classified as postoperative complication. The complications are listed in Table 4 [16,17,18,19,20,22,23,24] and Figure 4C. During regular outpatient follow-up, three patients had a recurrence and the transnasal endoscopic approach was used for one of them, as mentioned above. All patients with malignant tumors received chemoradiotherapy after surgery. Two patients died during follow-up; one patient survived for 3 years, while the other survived for 1 year and 7 months.

**Table 1:** Main symptoms and signs.

	Hughes et al. 1995 [17] *n* = 172	Cohen et al. 2005 [18] *n* = 166	Sun et al. 2017 [19] *n* = 103	Tao et al. 2018 [16] *n* = 188	Zhao et al. 2020 [20] *n* = 214	Our Center Cases *n* = 177
*Symptoms*						
External or intraoral mass	145	-	-	61	-	114
Otalgia	62	-	-	-	-	1
Dysphagia	22	12	10	-	-	3
Dysphonia	18	12	3	2	-	-
Dyspnea	-	1	1		-	2
Pain (facial)	11	10	-	6	-	-
Hearing loss	19	6	1	33 ^d^	21 ^f^	-
Foreign body sensation	-	-	12	102	143 ^g^	15
Tinnitus	3	8	-	33	-	4
Facial muscle weakness	7	-	-		-	-
Trismus	-	-	-	2	-	-
Painful throat	4	-	-	17	-	6
Tongue parasthesias	3	-	-	1	-	1
Aspiration	2	-	-	-	-	-
Headaches	2	-	-	2	5	1
Free symptoms	-	42	45	24	11	19
Snoring	-	1	8	15	15	3
Other	3 ^a^	3 _b_	23 ^c^	24 ^e^	19 ^h^	14 ^i^
*Signs*						
Intraoral mass	113	42	9	101	156	120
External mass	99	51	65	25	36	31
Cranial nerve deficit	22	38	5	6	11	4
Palatal weakness	9	14	-	-	-	-
Pulsation over mass	19	-	-	-	-	-
Hearing loss	15	-	-	-	-	-
Horner’s syndrome	3	3	-	-	-	-
Trismus	3	-	-	1	-	-
Shoulder weakness	-	7	-	-	-	-
Serous otitis media	-	6	-	-	-	-
Other unspecified	-	1	24	33	11	22

^a^ Lightheadedness; Hypertension; ^b^ Choking; Coughing; Nasal regurgitation; ^c^ Pain Hoarseness Rhinocleisis; ^d^ Hearing loss; Ear fullness; Tinnitus; ^e^ Hoarseness Rhinocleisis Epistaxis Face/neck parasthesias; Tongue pain; ^f^ Hearing loss; Ear fullness; Tinnitus; ^g^ Foreign body sensation; Painful throat; Pharyngeal itching; Intraoral mass; Dysphagia; ^h^ Hoarseness; Rhinocleisis; Epistaxis; ^i^ Ear fullness; Dizziness; Hoarseness; Facial parasthesias; Patients may have more than one symptom.

**Table 2:** Surgical approach.

Approach	Hughes et al. 1995 [17] *n* = 172	Shahab et al. 2005 [21] *n* = 114	Cohen et al. 2005 [18] *n* = 166	Zhi et al. 2009 [22] *n* = 162	Hong et al. 2015 [23] *n* = 112	Sun et al. 2017 [19] *n* = 103	Tao et al. 2018 [16] *n* = 188	Lombardi et al. 2020 [24] *n* = 153	Zhao et al. 2020 [20] *n* = 214	Our Center Cases *n* = 177
Cervical	49	49	89	51	51	75	159	49	167	131
Parotid	56	27	-	-	-	11	-	-	-	-
Cervical-parotid	63	-	20	93	45	-	8	56	-	11
Mandibular split	-	27	3	18	10	9	8	4	8	3
Transoral	2	-	-	-	6	8	7	8	23	20
Transoral–cervical	-	-	3	-	-	-	-	3		3
Transnasal	-	-	-	-	-	-	9	1	5	6
Infratemporal fossa	-	3	30	-	-	-	4	8	4	-
Other	-	8	-	-	-	-	-	22	-	3

The number of approaches does not add up to the number of patients due to: (1) combination of approaches; (2) underwent multiple procedures; (3) not receive surgical management.

**Table 3:** Histopathology.

Histopathology	Pensak et al. 1994 [27] *n* = 123	Hughes et al. 1995 [17] *n* = 172	Shahab et al. 2005 [21] *n* = 114	Cohen et al. 2005 [18] *n* = 166	Zhi et al. 2009 [22] *n* = 162	Hong et al. 2015 [23] *n* = 112	Sun et al. 2017 [19] *n* = 103	Tao et al. 2018 [16] *n* = 188	Lombardi et al. 2020 [24] *n* = 153	Zhao et al. 2020 [20] *n* = 214	Our Center Cases *n* = 177
Salivary gland lesions Benign											
Pleomorphic adenoma	-	68	34	33	57	39	16	61	53	36	26
Warthin’s tumour	-	1	-	-	3	10	-	-	3	-	1
Basal cell adenoma	-	-	-	-	5	-	-	7	-	3	1
Lymphoepithelial lesion	-	1	-	-	-	-	-	2	-		2
Myoepithelioma	-	-	-	1	-	-	-	-	-	3	1
Monomorphic adenoma	-	1	-	-	-	-	-	-	-	-	-
Granulomatous parotitis	-	-	-	-	-	-	-	-	-	-	-
Other/Unspecified	39	-	8	-	-	3	-	-	1	-	4
Malignant											
Adenoid cystic carcinoma	-	10	5	1	2	2	-	-	8	8	-
Mucoepidermoid carcinoma	-	-	3	1	3	4	-	-	2	5	3
Squamous cell carcinoma	-	3	-	-	1	-	-	-	-	27	1
Carcinoma ex pleomorphic adenoma	-	7	-	1	-	2	-	-	4	2	-
Adenocarcinoma	-	1	-	3	2	-	-	8	2	-	1
Acinic cell carcinoma	-	2	-	1	1	-	-	-	-	3	-
Myoepithelial carcinoma	-	-	-	3	-	-	-	-	1	3	-
Undifferentiated carcinoma	-	-	-	1	-	-	-	-	-	-	-
Other/Unspecified	12	-	4	-	-	-	-	-	4	-	1
Neurogenic lesions											
Benign											
Vagal paraganglioma	8	24	16	61	-	-	-	-	4	-	-
Carotid body tumour	9	9	17	2	-	4	8	-	-	-	-
Glomus jugulare	7	1	-	-	-	-	-	-	-	-	-
Sympathetic paraganglioma	-	-	-	4	-	-	-	-	-	-	-
Paraganglioma not specified	-	-	2	-	8	-	-	2	8	22	3
Schwannoma	6	-	11	16	36	20	34	77	12	39	88
Neurofibroma	2	-	3	7	18	9	4	1	1	4	7
Other/Unspecified	-	24	-	-	-	-	-	1	3	-	-
Malignant											
Unspecified malignant PNST ^a^	-	6	-	-	3	2	-	-	1	-	-
Malignant paraganglioma	-	-	-	2	3	-	-	-	1	-	-
Miscellaneous lesions											
Benign											
Aneurysm	2	-	-	6	4	-	-	-	-	-	-
Branchial cleft cyst	1	-	-	2	4	4	-	1	5	-	-
Hemangioma	1	1	-	1	5	-	5	10	2	2	5
Meninigioma	-	2	2	1	-	-	-	-	-	6	-
Lipoma	2	-	-	1	-	1	2	1	3	2	5
Inflammatory pseudotumour	4	-	-	-	-	-	5	3	-	-	-
Cystic hygroma	2	2	-	1	-	-	-	-	-	-	1
Other/Unspecified	-	2	5	7	4	3	14	2	4	8	16
Malignant											
Undifferentiated carcinoma	-	1	-	-	-	-	-	1	-	-	-
Chondrosarcoma	-	-	-	1	3	-	-	2	2	2	-
Sarcoma not specified	-	-	2	-	-	-	-	-	2	-	3
Hemangiopericytoma	3	-	-	1	-	-	-	-	-	1	-
Fibrosarcoma	1	-	-	-	-	-	-	-	-	1	-
Rhabdomyosarcoma	3	-	-	1	-	-	-	2	1	8	-
Chordoma	-	1	-	-	-	-	-	-	-	4	1
Malignant fibrous histiocytom	-	-	-	-	-	-	-	-	-	1	-
Other/Unspecified	1	2	-	1	-	-	9	3	2	11	-
Lymphoid lesions											
Lymphoma	4	3	2	-	4	-	4	4	1	1	1
Lymphoid hyperplasia	-	-	-	-	-	-	-	-	-	1	3
Reactive lymphoid tissue	-	-	-	-	-	-	-	-	-	2	-
Castleman’s disease	-	-	-	1	-	-	-	-	-	-	1
Other/Unspecified	-	-	-	-	-	8	-	-	-	-	-
Metastatic lesions											
Metastatic thyroid carcinoma	-	-	-	4	-	-	-	-	4	-	-
Metastatic squamous cell carcinoma	-	-	-	-	-	-	-	-	8	-	-
Other/Unspecified	11	-	-	1	-	1	-	-	11	-	-

^a^ Peripheral nerve sheath tumor (PNST).

**Table 4:** Postoperative complications.

Complications	Hughes et al. 1995 [17] *n* = 172	Cohen et al. 2005 [18] *n* = 166	Zhi et al. 2009 [22] *n* = 162	Hong et al. 2015 [23] *n* = 112	Sun et al. 2017 [19] *n* = 103	Tao et al. 2018 [16] *n* = 188	Lombardi et al. 2020 [24] *n* = 153	Zhao et al. 2020 [20] *n* = 214	Our Center Cases *n* = 177
Vth CN injury	-	-	-	-	-	-	6	-	-
VIIth CN injury	19	19	7	25	2	5	39	18	6
Xth CN injury	41	45	-	1	6	8	19	36	12
IXth CN injury	4	-	-	-	-	-	14	-	-
XIth CN injury	12	-	-	-	-	-	1	-	-
XIIth CN injury	15	15	-	3	1	3	7	13	6
Horner’s syndrome	11	12	5	2	3	4	10	11	2
First bite syndrome	-	18	-	-	-	-	6	-	-
Shoulder weakness	-	18	-	-	-	-	-	-	-
Trismus	-	6	-	-	-	-	-	-	3
Heamatoma	-	2	-	-	-	-	-	-	-
Vascular injury	6	-	-	-	1	4	8	2	2
Dysphagia	-	-	-	-	-	-	-	-	-
Dysphonia	-	-	8	-	-	-	-	-	-
Palatal insufficiency	-	33	-	-	-	-	-	-	-
Frey’s syndrome	-	-	-	4	-	-	8	-	-
Other	-	10	-	4	1	4	20	-	-

## 4. Discussion

The incidence of PPS tumors in the head and neck ranges from 0.5% to 1%, with most being benign tumors; pleomorphic adenoma and schwannoma are the most common [3]. A total of 175 patients underwent surgery in this study and more than 90% of the tumors were benign, which is consistent with previous reports [4]. Pleomorphic adenoma and schwannoma accounted for half of all benign tumors. These results are consistent with those of previous studies [20,21,22,23,24]. The most common symptoms for PPS lesions were external or intraoral masses and pharyngeal foreign body sensation, which is consistent with other studies [16,17]. Swelling of the neck and oropharynx was the most common sign, also reported in all large-sample studies [16,17,18,19,20]. Symptoms directly related to the tumor mass, including dysphagia, dyspnea, and dysphonia, were also found in the present study, similar to previous studies [18,19].

Owing to the deep position of the PPS and the slow growth of benign tumors, tumors tend to be large when diagnosed [28]. In this cohort, most lesions were located in the middle and upper portions. Therefore, the most common clinical symptoms of the patients were neck or intraoral masses. Physical examination confirmed intraoral asymmetry with protrusion in the patients. The proportion of our cohort with an asymmetrical mass in the neck or mouth was higher than that of previous studies [16,19]. This may be due to the late timing of patient visits. The Anhui region is relatively underdeveloped with respect to the economy and medical services, and patients are less willing to apply for medical assistance. This phenomenon is particularly prominent among patients from rural areas [29,30].

Considering the complexity of the anatomical and adjacent structures, surgical approaches vary. The transcervical approach allows direct access to the middle and inferior portions of the PPS, permitting satisfactory visualization of the cranial nerves and vessels. However, it may not be suitable for malignant tumors in the superior part, especially those adjacent to the skull base. The transcervical-parotid approach is mainly used for tumors close to the deep lobe of the parotid gland or tumors involving the facial nerves. The mandibular split method is commonly used for malignant, recurrent, or large tumors involving the internal carotid artery and the skull base. The indication for the infratemporal fossa approach is tumors involving the skull base or jugular foramen [31]. Due to the development of endoscopy, transoral or transnasal surgery is increasingly used for PPS lesions, given the advantages of reduced trauma and shorter duration of recovery [32]. However, it should be noted that this procedure is for highly selective cases and skillful surgeons. There is no unified conclusion on the choice of surgical approach, which depends more on the size or location of the tumor, histopathology, and experience of the surgeon. At our center, the transcervical approach is the most widely used. This procedure accounts for approximately 50% of all surgical procedures performed. The transcervical parotid approach is often the second choice when the isolated transcervical approach is not suitable. Compared with our samples, the transcervical-parotid approach was the most frequently used in the studies by Hughes et al. [17] and Zhi et al. [22]. In our experience, we extend the preauricular parotid incision to the neck for a wider field of vision. The superficial lobe of the parotid gland was displaced to protect the facial nerve. Finally, the lesion, extending from the deep lobe of the parotid gland to the PPS, was removed. This method can also be used to manage lesions located in the post-styloid space by removing the styloid process to allow better exposure of the upper portion of the PPS [33]. Carrau et al. [34] reported that prognathic dislocation of the mandible following stylomandibular ligament and styloid muscle division could widen the exposure by more than 50%. For lesions close to the internal carotid artery or larger malignant tumors, the maxilla is an obstacle to the complete removal of the tumor, as it limits exposure to the PPS. Mandibulectomy can be performed simultaneously in such patients. The indications for mandibulectomy mentioned in previous literature are few, and experts can avoid it in most cases [35,36]. During the early stages of our study, three patients underwent this procedure. One patient was treated with a combination of fracture and fixation of the mandibular ramus to expose the infratemporal fossa and part of the middle cranial fossa. Two patients underwent a partial resection of the posterior edge of the mandibular ramus. The main reason for this was that the tumor was still covered by the mandible after the mandibular ramus fracture. These patients developed postoperative trismus. As the mandible is involved, tracheotomy and nutrient tube placement are routinely performed in these patients after surgery. This is accompanied by upper airway obstruction and eating difficulties, which are almost always present in the short term after surgery. The procedure was traumatic, and postoperative complications significantly affected the patient’s life. Our center has tried to avoid this procedure since then.

Owing to their magnification and excellent aesthetics, endoscopes have gradually become important for head and neck surgery. However, endoscopy has limitations for use for PPS tumor resection [7]. Currently, it is only suitable for selected cases and requires endoscopic skills. All patients in our cohort who were operated on using the transoral approach underwent endoscopy. Endoscopy can mitigate the problems associated with visual field and facilitate quick recovery associated with reduced trauma. Earlier attempts were aimed at benign tumors in the anterior styloid process. With technology, it has been reported in recent literature that the transoral approach involving the posterior styloid space has been completed safely [37,38]. Although the transoral approach has several advantages, its disadvantages, such as very limited visual field exposure, are significant. Narrow anatomical pathways may present constraints around the tumor and make it difficult to control bleeding. The transoral approach may involve the soft palate muscle, and the risk of damage to the vagus nerve branches persist. Postoperative patients often require short-term fasting to prevent postoperative infections [7]. At present, research on the long-term advantages of the oral approach over the cervical approach is very limited, and more research is needed to verify this in the future. The endoscope-assisted approach can be used to overcome the limitations of some cervical approaches, especially when the tumor is located at the superior part of the PPS. Endoscopy guidance during the transcervical approach facilitates the identification of surgical landmarks, provides anatomical guidance, and facilitates hemostasis [39,40]. All of these factors help decrease surgical complications.

In our study, 31 patients (18%) developed postoperative complications. The most common postoperative complications were injuries of cranial nerves VII and X, which manifest as facial paralysis and hoarseness. This result is consistent with those of previous studies [16,17,18,19,20,23,24,41]. The intracordal injection is an alternative method for treating this complication [42,43]. Patients with vocal cord paralysis in our group tolerated the complications well; therefore, no other surgical intervention was performed. Another important complication is the first bite syndrome, which is described as pain in the parotid region caused by the first bite of a meal. It is associated with sympathetic injury to the parotid gland [44]. Studies have reported that botulinum toxin A injection in the parotid gland area, as a safe and noninvasive treatment, can decrease the severity of this symptom [45]. However, no first bite syndrome was observed in the present study. Facial paralysis occurs in patients using the transcervical-parotid approach. In addition to facial nerve resection for malignant tumors, the symptoms of facial paralysis in other patients improved during the follow-up period after surgery, and were classified as grade II-III according to the House-Brackmann grading system [46].

The recurrence rate of PPS tumors is low [4]. In this group, there were three cases of recurrence. Considering the risk associated with the scar tissue and structural damages after the initial surgery, we adopted the endoscopic transnasal approach to remove recurrent lesions. The recurrence rate in the current study was lower than that in other studies. First, there were fewer malignant tumors in the current cohort, and second, some patients sought additional interventions [15]. Malignant tumors generally have greater recurrence rates than benign tumors, as in PPS tumors. A previous study reported about 50% mortality rate of malignant tumors in PPS; therefore, regular postoperative follow-up is still necessary [19].

## 5. Conclusions

PPS tumors are rare, and they present with atypical clinical manifestations. Surgery is the first choice of treatment. The optimal procedure choice should be individualized based on availability, patient characteristics, and the surgeon’s experience. Endoscopy assisted surgery combined with an extraoral approach may be a better choice, which can help expand the scope of surgery and reduce postoperative complications.

## Data Availability

Data can be obtained by scientists that work independently from the industry on request. Data are not stored on publicly available servers.
